# AphA-dependent c-di-GMP production in *Vibrio parahaemolyticus* is mediated by direct regulation of *eapA* transcription encoding an EAL domain-containing protein

**DOI:** 10.1128/jb.00104-25

**Published:** 2025-09-18

**Authors:** Nan Zhang, Wu Xu, Xue Li, Miaomiao Zhang, Xi Luo, Bin Ni, Renfei Lu, Yiquan Zhang

**Affiliations:** 1 Department of Laboratory Medicine, School of Medicine, Jiangsu University 12676, Zhenjiang, Jiangsu, China; 2 Department of Clinical Laboratory, Nantong Third People's Hospital, Affiliated Nantong Hospital 3 of Nantong University 66479, Nantong, Jiangsu, China; Geisel School of Medicine at Dartmouth, Hanover, New Hampshire, USA

**Keywords:** *V. parahaemolyticus*, AphA, c-di-GMP, biofilm formation, motility, regulation

## Abstract

*Vibrio parahaemolyticus,* a major seafood-borne pathogen, employs quorum sensing (QS) and c-di-GMP to regulate virulence, motility, and biofilm formation. While the master QS regulator AphA promotes c-di-GMP accumulation at low cell density (LCD), the underlying mechanism remained unclear. Here, we show that AphA drives net c-di-GMP accumulation by elevating c-di-GMP production while also activating the transcription of *eapA* (*vp0376*), encoding an EAL domain-containing phosphodiesterase, revealing a complex regulatory node. RNA sequencing revealed that AphA regulates 1,542 genes, including 23 potentially linked to c-di-GMP metabolism. Among these, *eapA* exhibited the strongest predicted AphA-binding motif. Experimental validation confirmed AphA binds the *eapA* promoter to activate its transcription. The expression of *eapA* peaked at LCD and decreased with increasing cell density. Deletion of *eapA* elevated c-di-GMP levels at LCD, enhanced biofilm formation, and impaired swimming motility, while *aphA* deletion reduced c-di-GMP. The *aphA*- and *eapA* double mutant exhibited c-di-GMP and biofilm phenotypes resembling the *eapA* mutant, placing EapA downstream of AphA. Transcriptional analysis showed *eapA* deletion upregulated exopolysaccharide biosynthesis genes while downregulating polar flagellar genes, aligning with c-di-GMP-mediated biofilm-motility trade-offs. Our findings establish a direct AphA-EapA-c-di-GMP pathway that critically regulates the biofilm-motility switch in *V. parahaemolyticus*, revealing how QS integrates with second-messenger signaling to optimize environmental adaptation.

**IMPORTANCE:**

*Vibrio parahaemolyticus* (*V. parahaemolyticus*) poses significant threats to human health and aquaculture, yet the mechanisms linking QS to c-di-GMP signaling remain poorly understood. This work uncovers AphA as a pivotal regulator that directly activates *eapA*, an EAL domain phosphodiesterase (PDE), to elevate c-di-GMP levels at low cell density (LCD). We identify EapA as the LCD-specific PDE that degrades c-di-GMP and is directly activated by AphA. Deletion of *eapA* elevates c-di-GMP levels, enhancing biofilm formation while suppressing swimming motility; these phenotypes are epistatic to AphA. The discovery of the AphA-*eapA*-c-di-GMP axis provides novel insights into how QS integrates with second messengers to optimize bacterial fitness. This study underscores the complexity of c-di-GMP metabolism and highlights AphA’s dual role as a global transcriptional regulator, bridging gaps in our understanding of bacterial signaling networks.

## INTRODUCTION

*Vibrio parahaemolyticus*, a gram-negative, halophilic bacterium widely distributed in marine ecosystems, is the leading cause of seafood-related acute gastroenteritis, characterized mainly by diarrhea, abdominal pain, vomiting, and fever (1, 2). In rare cases, *V. parahaemolyticus* causes wound infections, leading to necrotizing fasciitis or cellulitis (3). Additionally, it induces acute hepatopancreatic necrosis disease in marine animals such as shrimp, posing significant risks to global aquaculture (4). The pathogenicity of *V. parahaemolyticus* relies on diverse virulence factors, including lipopolysaccharides, hemolysins, type III secretion systems (T3SSs), extracellular proteases, type VI secretion systems (T6SSs), and iron acquisition systems, each contributing distinct physiological roles during infection (2).

*V. parahaemolyticus* is commonly found in a biofilm state in natural environments. Biofilms are structured bacterial communities attached to surfaces and embedded within an extracellular matrix, which primarily consists of exopolysaccharides (EPS), lipids, proteins, and extracellular (eDNA) (5). Among these components, EPS is the most critical, as it constitutes the main structural element of the biofilm matrix (6). In *V. parahaemolyticus*, the gene clusters *cpsA-K* and *scvA-O* are responsible for EPS synthesis, and both promote biofilm formation (7, 8). Additionally, flagella-mediated motility is critical for biofilm formation (9). *V. parahaemolyticus* possesses two flagellar systems: a polar flagellum for swimming in liquid environments and lateral flagella for swarming on surfaces (10). Strains lacking a polar flagellum lose the ability to form mature biofilms, whereas exogenous polar flagellin enhances biofilm formation in *V. parahaemolyticus* (11, 12). Biofilm formation is regulated by various signaling molecules and regulatory systems, with c-di-GMP and the quorum sensing (QS) system serving as two crucial components of the biofilm regulatory network (13).

c-di-GMP acts as a ubiquitous bacterial second messenger that post-transcriptionally controls processes such as virulence, motility, and biofilm development (14). Its cellular levels are balanced by diguanylate cyclases (DGCs) with GGDEF domains (which synthesize c-di-GMP) and phosphodiesterases (PDEs) containing EAL or HD-GYP domains (which degrade c-di-GMP) (14). In *V. parahaemolyticus*, the first identified PDE is ScrC, a dual-domain (GGDEF/EAL) protein encoded by the *scrABC* operon, which exhibits context-dependent activity (15). ScrC exhibits PDE activity to degrade c-di-GMP in the presence of ScrA and ScrB but functions as a DGC to synthesize c-di-GMP in their absence (15). Similarly, ScrG (16) and GepA (17) possess dual GGDEF/EAL domains but demonstrate only PDE activity, inhibiting biofilm formation and promoting motility. GGDEF-domain-containing proteins VPA0198 (18), ScrJ (19), ScrL (19), GefA (20), and GefB (21) are active DGCs in *V. parahaemolyticus* that promote c-di-GMP synthesis, enhance biofilm formation, and/or suppress motility. TpdA, an EAL-domain-containing protein, degrades c-di-GMP in planktonic cells (22). VopY, another EAL-domain-containing protein, hydrolyzes c-di-GMP and supports T3SS2-dependent enterotoxicity and cytotoxicity (23). Despite progress in characterizing c-di-GMP metabolic enzymes in *V. parahaemolyticus*, the roles of many putative genes encoding these enzymes remain poorly characterized.

QS is a cell-to-cell signaling mechanism used by bacteria to regulate gene expression and behaviors in response to fluctuations in environmental autoinducer concentrations (24). This system controls gene expression through master regulators (24, 25). In *V. parahaemolyticus*, AphA and OpaR serve as the master regulators of the QS system under low cell density (LCD) and high cell density (HCD) conditions, respectively (26, 27). At LCD, AphA activates key virulence genes, flagellar genes, and biofilm-formation-related genes (24). Conversely, at HCD, OpaR replaces AphA to regulate these processes but exerts opposing effects (28–30). AphA and OpaR also modulate c-di-GMP metabolism in *V. parahaemolyticus* (29, 31). Under surface-growth conditions, OpaR inhibits c-di-GMP production by regulating a group of proteins containing GGDEF and/or EAL domains (29). However, in liquid culture, OpaR enhances c-di-GMP levels by repressing *tpdA* transcription (22). Similarly, AphA promotes c-di-GMP synthesis at LCD, though the underlying mechanisms remain poorly characterized (31).

In this study, we found that AphA promotes c-di-GMP production and directly activates the transcription of *vp0376* encoding an EAL domain-containing protein that functions under LCD conditions, revealing an unexpected role in PDE regulation. Furthermore, VP0376 inhibits biofilm formation and enhances swimming motility. As VP0376 represents the first EAL domain-containing PDE identified by our group, we have designated it EapA (EAL-containing phosphodiesterase A).

## MATERIALS AND METHODS

### Bacterial strains

*V. parahaemolyticus* RIMD2210633 (wild type, WT) was used as the parental strain in this study (32). The non-polar *aphA* gene deletion mutant (Δ*aphA*) was constructed in a previous study (26). The *eapA* deletion mutant (Δ*eapA*) and *aphA* and *eapA* double-gene mutant (Δ*aphA*Δ*eapA*) were generated by removing a 423 bp fragment (nucleotides 63–485) of the *eapA* coding sequence from the WT strain and the Δ*aphA* strain, respectively, via homologous recombination using the suicide plasmid pDS132 (28). For complementation, the *eapA* coding region along with a ribosome-binding site (AGGAGG) was cloned into the *Sma*I and *Sal*I sites of pBAD33, a vector harboring an l-arabinose-inducible promoter and a chloramphenicol resistance marker (33). The resulting plasmid, pBAD33-*eapA*, was introduced into the Δ*eapA* strain to generate the complemented strain Δ*eapA*/pBAD33-*eapA* (C-Δ*eapA*). Control strains were generated by introducing the pBAD33 into the WT, Δ*eapA,* and Δ*aphA*Δ*eapA* backgrounds, yielding WT/pBAD33, Δ*eapA*/pBAD33, and Δ*aphA*Δ*eapA*/pBAD33, respectively. All primers are listed in Table 1.

**TABLE 1:** Primers used in this study

Target	Primers (forward/reverse, 5′−3′)	Reference
Construction of mutant		
*eapA*	GCGCCTGCAGTGAGACACCAAGAGAAAACG/CCCCATCAATAACACCTAGTACGCAACTTCGCTCACTCCT	This study
	AGGAGTGAGCGAAGTTGCGTACTAGGTGTTATTGATTGGG/GCGCGCATGCAACCAAGCATAACTAACAGC	
	GCGCCTGCAGTGAGACACCAAGAGAAAACG/GCGCGCATGCAACCAAGCATAACTAACAGC	
Construction of complementation strain		
*eapA*	GCGGGATCCAGGAGGAATTCACCATGTTTACTGTTGAATACTC/GCGAAGCTTTTATAGATTCATTTGTAGCA	This study
RT-qPCR		
*eapA*	ACTACGAGGCATTATCAAGC/CATTTGCAGAAGCAATACAG	This study
*cpsA*	GAGAGCGGCAACCTATATC/GCGGTCAAACAAAGGGTAAAC	(30)
*scvE*	GACAGGTCGTGATGCCATTC/GGCGATGATGACCGAAGTG	(30)
*flgM*	ATTCAAGTGCGACATCAAG/CGGAGAAGCTGCCATATC	(17)
*flgB*	ACAAGGCACTAGGCATCC/GACCATCTGTTCGGCTAAG	(17)
*flaC*	GGCTGAAGGTGCGATGAAC/AGACGACGACCACCGAATG	This study
*flaD*	CTTCGGGTTTCAAAATCAACAGC/TTGCGAACAGCCACATCCAG	This study
*flaF*	TGGCTATCACCGTTAATACC/CGCTGTTAATACGCTTCC	This study
*flaB*	ACAACGTATGCGTGACCTGT/TCAGCACCGATTTGGAACGA	This study
*recA*	GCTAGTAGAAAAAGCGGGTG/GCAGGTGCTTCTGGTTGAG	(34)
LacZ fusion/two-plasmid LacZ fusion		
*eapA*	GCGCGTCGACAGAATGTGATCGCCAGTTTGC/GCGCGAATTCATCGTTGTCCTACCGCCTTC	This study
Luminescence assay		
*eapA*	GCGCGAGCTCAGAATGTGATCGCCAGTTTGC/GCGCGGATCCATCGTTGTCCTACCGCCTTC	This study
*aphA*	GCGCACTAGTACCATTCGTAATACAAAAGGC/GCGCACTAGTGCTTTCCAGAAGTAACCGA	This study
EMSA		
*eapA*	AGAATGTGATCGCCAGTTTGC/ATCGTTGTCCTACCGCCTTC	This study
DNase I footprinting		
*eapA*	CGTAGCGGATTCTGTATTTGA/CCCGTCATTACCAAGCAATG	This study
5′-RACE		
*eapA*	GCTGATGGCGATGAATGAACACTG/ATCGTTGTCCTACCGCCTTC	This study
	GAACACTGCGTTTGCTGGCTTTGATG/GTTGCGATCCTACACTTGCG	

### Growth conditions

For bacterial culture, 10 µL of glycerol stock was inoculated into 5 mL 2.5% (wt/voll) Heart Infusion (HI) broth (BD Biosciences, USA) in 2 cm diameter glass test tubes and incubated at 37°C with shaking for 12 h. The culture was then diluted 1:50 into 5 mL HI broth and incubated under identical conditions until the optical density at 600 nm (OD_600_) reached 1.4, designated as the bacterial seed. This seed was further diluted 100-fold into 10 mL fresh HI broth for a third round of growth. Bacterial cells were collected at specified cell densities as required. For strains carrying pBAD33, the medium was supplemented with 2.5 µg/mL chloramphenicol (or 20 µg/mL for *Escherichia coli* strains) and 0.1% l-arabinose to induce gene expression. For strains carrying pBBRlux, 5 µg/mL chloramphenicol was added to the medium. For strains containing pBRP309, gentamicin (100 µg/mL) was included in the medium.

### Growth curves

The bacterial seed was diluted 1,000-fold into 10 mL HI broth, mixed thoroughly, and then divided into a 96-well plate with 200 µL per well. Each strain was tested with 10 technical replicates. Growth curves were measured using a microbial growth curve analyzer (MGC-200, Ningbo Scientz Biotechnology, China) by measuring OD_600_ values at 30-min intervals (21). Bacteria were cultured at 37°C with shaking at 800 rpm.

### RNA sequencing (RNA-seq)

Bacterial cells of the WT and Δ*aphA* strains were harvested in triplicate at OD_600_ values of 0.15 to simulate LCD growth conditions (26). Total RNA was prepared using TRIzol Reagent (Invitrogen, USA). RNA quality assessment, mRNA enrichment, cDNA library preparation, and sequencing were performed by GENEWIZ Biotechnology (Suzhou, China). Gene expression in the Δ*aphA* strain was analyzed relative to the WT strain. Differentially expressed genes (DEGs) were analyzed using DESeq (v1.12.4) with a significance threshold of *P* ≤ 0.01 and an absolute fold change ≥2. Functional analysis of DEGs was performed using gene ontology (GO), Kyoto Encyclopedia of Genes and Genomes (KEGG), and Clusters of Orthologous Groups (COG) databases (35). GO enrichment analysis used Goseq (36), which employs the Wallenius non-central hypergeometric distribution to account for gene length bias, providing more accurate probabilities of GO term enrichment. Significantly enriched GO terms were defined by an over_represented_*P* ≤ 0.05. KEGG pathway enrichment analysis utilized the hypergeometric test to identify pathways significantly enriched in DEGs compared to the whole genome background (37). This identifies key metabolic and signaling pathways involving DEGs. COG analysis involved annotating and classifying DEGs within the COG database (NCBI), which groups homologous proteins across species. This predicts protein function and identifies the primary functional categories represented among the DEGs.

### Real-time quantitative PCR (RT-qPCR)

Total RNA was extracted from bacterial cells harvested at OD_600_ ≈ 0.15. cDNA was generated from 1 µg of total RNA with the FastKing First Strand cDNA Synthesis Kit (Tiangen Biotech, China). RT-qPCR was performed on a LightCycler 480 system (Roche, Switzerland) using SYBR Green-based PCR master mix (Tiangen Biotech, China). Relative expression levels of target genes were calculated via the 2^−ΔΔC*t*^ method, with *recA* serving as the internal reference gene (18, 34).

### Luminescence assay

The luminescence assay was conducted according to a previously described method (21). The promoter DNA region of *eapA* or *aphA* was inserted into the pBBRlux vector containing a promoterless *luxCDABE* operon. The recombinant pBBRlux vector was subsequently introduced into the WT strain to assess promoter activity driving the *luxCDABE* operon, as measured by luminescence. The relative luminescence units (RLU) were expressed as light units per OD_600_.

### LacZ fusion

The promoter DNA region of *eapA* was inserted into pHRP309, which was then transferred into *V. parahaemolyticus* strains (WT and Δ*aphA*) or *E. coli* 100 λpir (EC100) each harboring either the complementation pBAD33 plasmid (pBAD33-*aphA*) or the empty pBAD33 plasmid. The cells of *V. parahaemolyticus* and *E. coli* were harvested at OD_600_ ≈ 0.15 and 1.0, respectively, then lysed to prepare cellular extracts. β-galactosidase in the extracts was measured using the β-Galactosidase Enzyme Assay System (Promega, USA). Miller Units, representing β-galactosidase activity, were calculated (30).

### Preparation of 6× His-tagged AphA (His-AphA) protein

The recombinant pET28a encoding His-AphA was constructed in our previous study and transformed into *E. coli* BL21λDE3 for His-AphA expression (26). Purification of His-AphA was performed as previously described (26). The purity of His-AphA was confirmed by sodium dodecyl sulfate-polyacrylamide gel electrophoresis. The purified His-AphA was stored at −60°C until use.

### Electrophoretic mobility-shift assay (EMSA)

The 5′-ends of the promoter DNA fragments of *eapA* were labeled with [γ-^32^P] ATP for EMSA (28), which was performed in a 10 µL reaction volume containing binding buffer (1 mM MgCl_2_, 0.5 mM DTT, 50 mM NaCl, 0.5 mM EDTA, 10 mM Tris-HCl pH7.5, and 0.05 mg/mL salmon sperm DNA), DNA probe (1,000–2,000 CPM/µL), and His-AphA. Three controls were included: (i) specific DNA competitor (unlabeled promoter DNA region of *eapA*), (ii) nonspecific DNA competitor (unlabeled 16S rDNA), and (iii) rabbit anti-F1-protein polyclonal antibodies for nonspecific protein competitor. Binding products were resolved in a native 4% (wt/vol) polyacrylamide gel and analyzed by autoradiography.

### DNase I footprinting assay

DNase I footprinting assay was performed as previously described (28). DNA binding was performed in a reaction system similar to that of EMSA. Reactions were incubated at 25°C for at least 20 min. Prior to digestion, 10 µL of Ca^2+^/Mg^2+^ solution was added and incubated at 25°C for another 1 min. RNase-Free DNase I (Promega, USA) was added to the reaction mixtures, followed by incubation at 25°C for 40–90 s. Reactions were quenched by adding 9 µL of stop solution (200 mM NaCl, 30 mM EDTA, and 1% SDS). Digested DNA samples were extracted using phenol-chloroform, ethanol-precipitated, and analyzed on a 6% polyacrylamide/8 M urea gel. Protected regions were identified by comparison with sequencing ladders (generated using the same DNA fragments as in the footprinting assays). Radiolabeled fragments were detected by autoradiography.

### 5′-RACE

Total RNA was extracted from WT cells harvested at OD_600_ ≈ 0.15. Contaminating DNA was digested using the FastKing RT Kit (With a gDNase) (Tiangen Biotech, China). Subsequently, the HiScript-TS 5′/3′ RACE Kit (Vazyme, China) was employed to amplify transcripts of the *eapA* gene by nested PCR, following the manufacturer’s protocol. Amplified products were cloned into the T-vector and sent to GENEWIZ Biotechnology (Suzhou, China) for sequencing to identify the transcription start site.

### c-di-GMP quantification

The c-di-GMP levels were measured as previously described (21). Briefly, bacterial cells were collected at OD_600_ ≈ 0.15 and 1.4, respectively, washed two times with cold phosphate-buffered saline (PBS), and resuspended in 0.6 mL of ice-cold PBS. The cells were sonicated for 15 min to extract the intracellular contents. After centrifugation, the supernatant was collected for c-di-GMP measurement and total protein quantification. c-di-GMP concentration was determined with the Enzyme-Linked Immunosorbent Assay (ELISA) Kit (Mskbio, China), and total protein was measured using the Pierce BCA Protein Assay kit (ThermoFisher Scientific, USA). c-di-GMP contents were expressed as picomoles per gram of protein.

### Crystal violet (CV) staining

The bacterial seed was diluted 1:2,000 into 10 mL of HI broth, vortexed, and divided into a 96-well plate (200 µL per well), with five replicates per strain. Cultures were incubated at 30°C with shaking 100 rpm for 24 h. OD_600_ values of the cultures were measured to assess bacterial growth. Biofilms were washed with deionized water, stained with 0.1% CV solution, and rinsed three times with deionized water to remove unbound CV dye. Bound CV was solubilized in 300 µL of 15% acetic acid, and biofilm mass was quantified by measuring OD_570_. Relative biofilm formation was calculated as the OD_570_:OD_600_ ratio (21).

### Scanning electron microscopy (SEM)

SEM was performed as previously described (38). Briefly, the bacterial seed was diluted 1:2,000 into 5 mL of HI broth, vortexed, and aliquoted into a 24-well plate containing sterile glass slides (2 cm × 1 cm × 0.2 mm), with 1 mL of suspension per well and three technical replicates per strain. Plates were incubated at 30°C and 150 rpm for 24 h for biofilm formation. After incubation, slides were removed, washed three times with PBS, fixed with 4% paraformaldehyde, then rinsed another three times with PBS, and then sequentially dehydrated in ethanol solutions (30%, 50%, 60%, 70%, 90%, 95%, and two times in 100% ethanol) for 10 min per step. Slides were air-dried, coated with gold-palladium using the Hitachi Ion Sputter MC1000 (Hitachi, Japan), and imaged with a field-emission SEM (HITACHI SU8010, Japan).

### Swimming and swarming motility assays

Swimming motility assays (39) were performed by inoculating 2 µL of bacterial seed into HI plates (7 cm in diameter) containing 0.2% Difco Noble agar (BD Biosciences, USA; five technical replicates per strain). Because *V. parahaemolyticus* swims rapidly, only one strain was inoculated in the middle position on each plate. Plates were incubated at 37°C, and the swimming zone diameter was measured after 7 h of incubation and photographed. Swarming motility assays (39) were conducted similarly, with 2 µL of bacterial seed inoculated onto HI plates (7 cm in diameter) containing 1.8% Difco Noble agar (five technical replicates per strain). The swarming speed of *V. parahaemolyticus* is relatively slow, so all strains were inoculated as separate positions on a single plate. Plates were incubated at 37°C for 36 h, and swarming zone diameter was measured and photographed.

### Statistical methods

All experiments (except RNA-seq) included at least three biological replicates, each with three technical replicates. Numerical data are presented as means ± standard deviation (SD). Statistical significance was assessed using two-way ANOVA followed by Tukey’s post hoc test. A *p* value < 0.05 was considered statistically significant.

## RESULTS

### Deletion of *aphA* alters global gene expression at LCD

**Fig 1:**
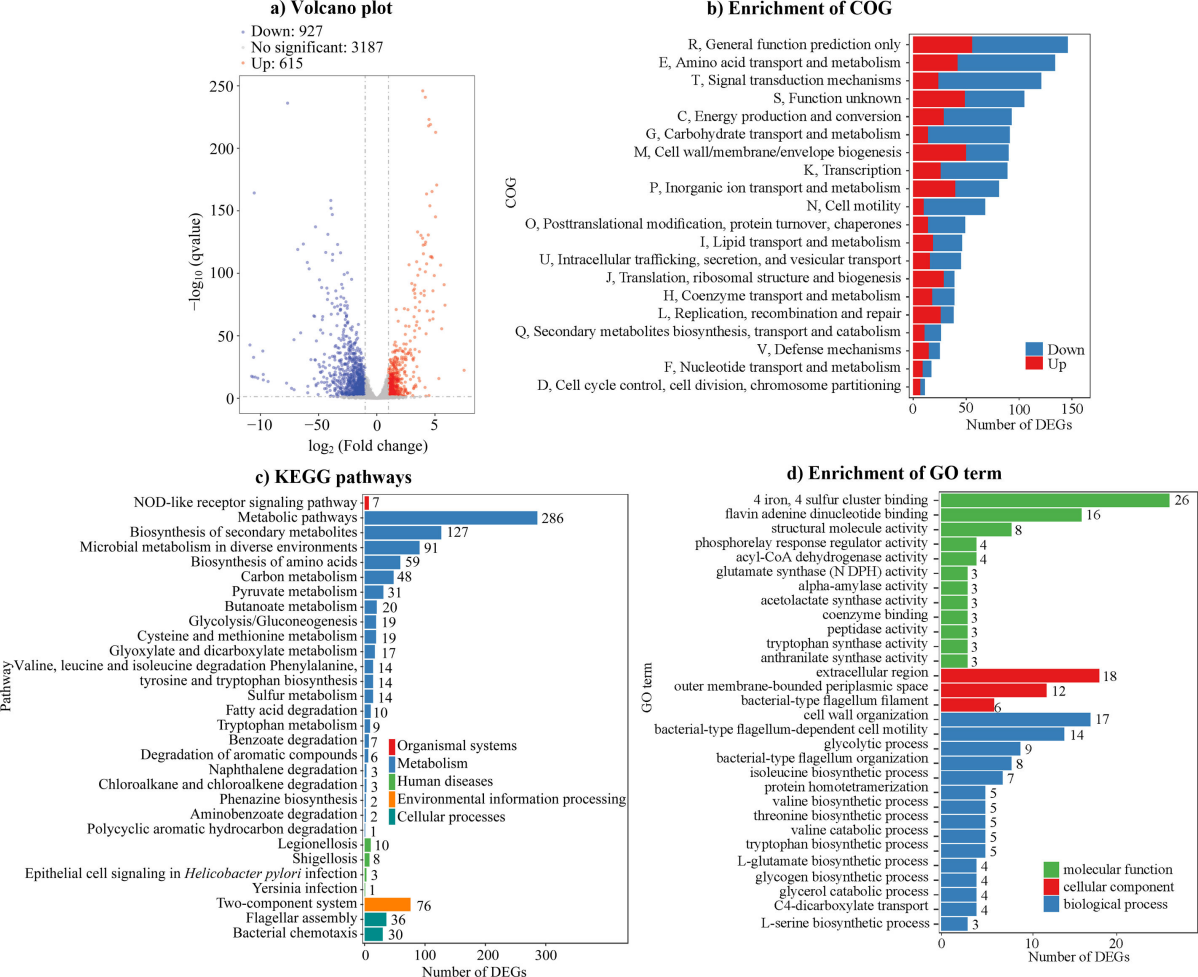
AphA regulates global gene expression at LCD. (a) Volcano plot of DEGs. Gray dots represent genes with unchanged expression levels, while blue and red dots indicate downregulated and upregulated DEGs in Δ*aphA* relative to WT, respectively. (b) COG enrichment. Red bars denote upregulated DEGs, and blue bars indicate downregulated DEGs. (c) KEGG pathway enrichment. (d) GO term enrichment. The numbers on the right side of the bars in (c) and (d) indicate the count of DEG in each category.

RNA-seq was conducted to identify the AphA regulon in *V. parahaemolyticus* under LCD conditions. Deletion of *aphA* significantly altered the transcription of 1,542 genes compared to the WT strain (Fig. 1a; Table S1). Among these DEGs, 927 were downregulated (indicating positive regulation by AphA), while 615 were upregulated (suggesting negative regulation by AphA). COG enrichment analysis classified the DEGs into 20 functional categories (Fig. 1b). The five most enriched categories were general function prediction only, amino acid transport and metabolism, signal transport and metabolism, function unknown, and energy production and conversion. KEGG pathway analysis revealed that the majority of DEGs (802 genes) were linked to metabolism, followed by 76 genes associated with environmental information processing, 66 with cellular processes, 22 with human diseases, and 7 with organismal systems (Fig. 1c). GO term enrichment categorized the DEGs into molecular functions (79 DEGs across 12 terms), cellular components (36 DEGs across 3 terms), and biological processes (94 genes across 15 terms) (Fig. 1d). Collectively, these findings highlight AphA as a global transcriptional regulator in *V. parahaemolyticus*, modulating diverse cellular pathways under LCD conditions.

### Identification of AphA-regulated c-di-GMP-associated genes

Among the DEGs, there are 23 c-di-GMP-related genes (17 downregulated and 6 upregulated), 42 polar flagellar genes (all downregulated), 5 lateral flagellar genes (all downregulated), 9 *scv* genes (all downregulated), 25 T3SS1 genes (24 downregulated and 1 upregulated), 11 CPS genes (all upregulated), 3 T6SS1 genes (all upregulated), 6 T6SS2 genes (all upregulated), 43 T3SS2 genes (all upregulated), and 104 regulator genes (85 downregulated and 19 upregulated) (Table S2). The regulatory effects of AphA on flagellar genes, *scv* genes, T3SS genes, and T6SS1 genes are consistent with the findings in previous studies (28, 40–43). A previous study has demonstrated that AphA positively regulates c-di-GMP metabolism, but the underlying mechanisms remain poorly characterized (31). Therefore, this study focused on the regulatory role of AphA in c-di-GMP metabolism.

The 500 bp upstream DNA sequences of the 23 c-di-GMP-associated genes were retrieved from the WT genome. Using the known DNA binding motif of AphA, predicted AphA binding sites within these regions were identified using the Matrix Scan tool (26, 44). This tool assigns weight scores to potential binding sites, where higher scores indicate a greater likelihood of direct AphA binding. Predicted AphA binding sites were detected only in the putative promoter regions of *eapA*, VP1979, VP2366, VPA0476, and VPA0518, but not in the other 18 genes (Table 2). Previous studies have consistently reported that DNA sequences experimentally confirmed to be directly bound by AphA possess weight scores exceeding 10 (26, 28, 42, 45). In this analysis, however, only the *eapA* upstream sequence achieved a weight score above this threshold. Therefore, *eapA* was selected for further investigation of AphA’s regulatory mechanism.

**TABLE 2:** AphA box-like sequences within target DNA sequences^*a*^

Gene	Strand	Start	End	AphA box-like sequence	Weight score
*eapA*	R	−281	−262	ATATGAAAAAATACACTTAT	10.6
VP1979	D	−188	−169	ATAATCAAATTAGTGCATAG	5.7
VP2366	R	−213	−194	ATAGTCGACTATTTGCATAT	8.1
VPA0476	D	−127	−108	TTAAGCAACCTGACACTCAC	4.8
VPA0518	D	−94	−75	ATAGTTTACTTGATGCATAT	5.5

^*a*^ “D” represents the direct sequence, while “R” indicates the reverse sequence. Negative numbers represent nucleotide positions upstream of the start codon.

### AphA directly activates *eapA* transcription

**Fig 2:**
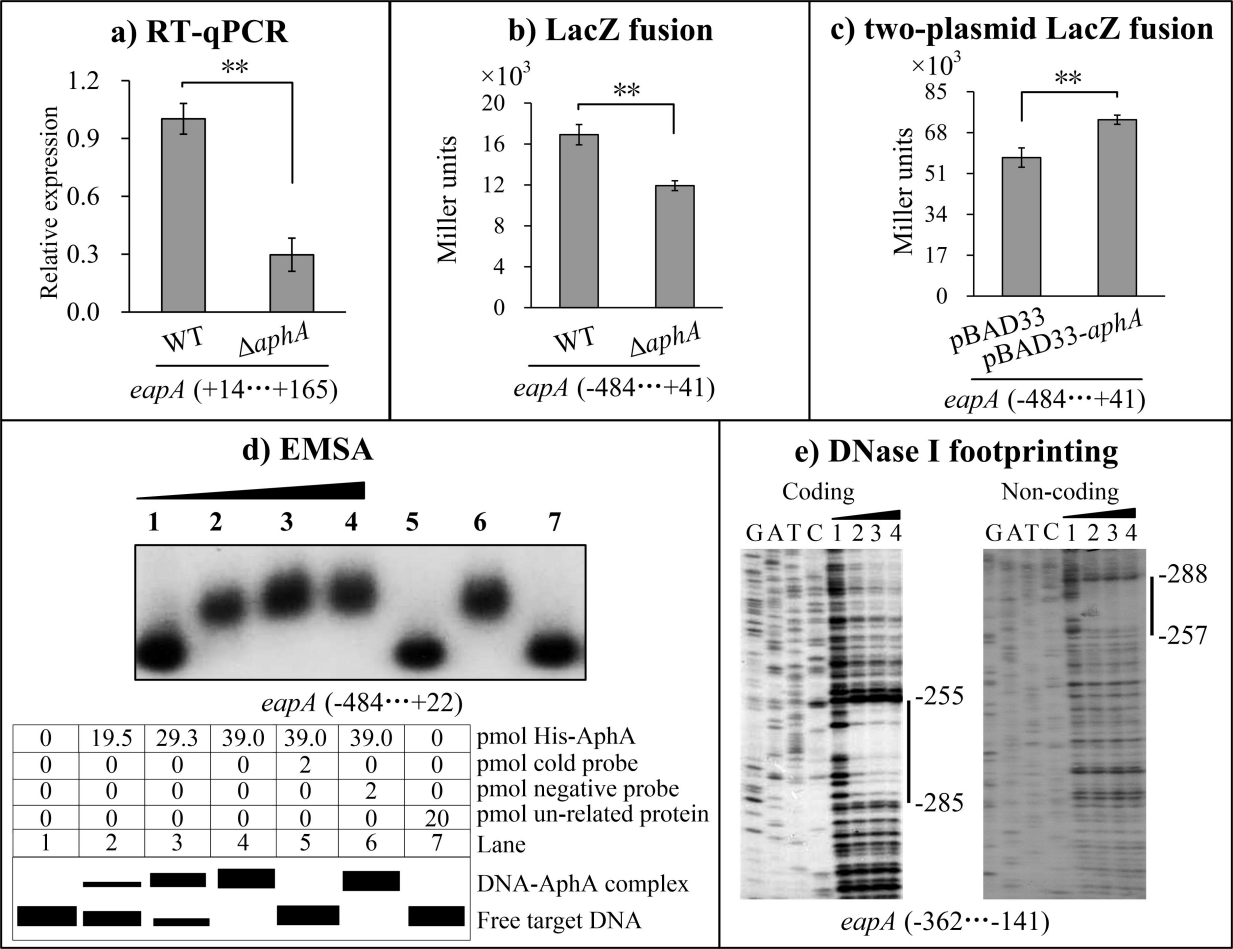
AphA directly activates *eapA* transcription*.* Negative and positive numbers indicate nucleotide positions upstream and downstream relative to the first nucleotide of the translation start codon (+1), respectively. **, *P* < 0.01. (a) RT-qPCR. Relative *eapA* mRNA levels in Δ*aphA* were compared with those in WT. (b) LacZ fusion. The promoter region of *eapA* was inserted into pHRP309 and transformed into WT and Δ*aphA*. β-galactosidase activity was measured in cell lysates. (c) Two-plasmid *lacZ* fusion. Assays were performed as in (b). (d) EMSA. The *eapA* promoter region was incubated with purified His-AphA and resolved by 6% (wt/vol) polyacrylamide gel electrophoresis. A schematic of EMSA design is shown below the gel image. (e) DNase I footprinting. DNA probes were incubated with purified His-AphA. Protected regions (vertical bars) were identified relative to Sanger sequencing lanes (G, A, T, C). Lanes 1–4 contained 0, 15, 20, and 25 pmol of His-AphA, respectively.

RT-qPCR revealed significantly reduced mRNA levels of *eapA* in the Δ*aphA* strain compared to the WT strain (*P* < 0.01; Fig. 2a). The *lacZ* fusion assay showed reduced *eapA* promoter activity in the *ΔaphA* strain relative to the WT strain (*P* < 0.01; Fig. 2b)*.* Using a two-plasmid *lacZ* fusion system, AphA overexpression in *E. coli* significantly induced *eapA* promoter activity (*P* < 0.01; Fig. 2c). EMSA results confirmed dose-dependent binding of His-AphA to the *eapA* upstream DNA fragment (Fig. 2d), consistent with bioinformatic predictions (Table 2). DNase I footprinting assay further mapped the His-AphA binding site to the region spanning positions −288 to −255 relative to the translation start site (+1). Collectively, these findings demonstrate that AphA directly activates transcription of *eapA*.

### Transcriptional start site and promoter-proximal structure of *eapA*

**Fig 3:**
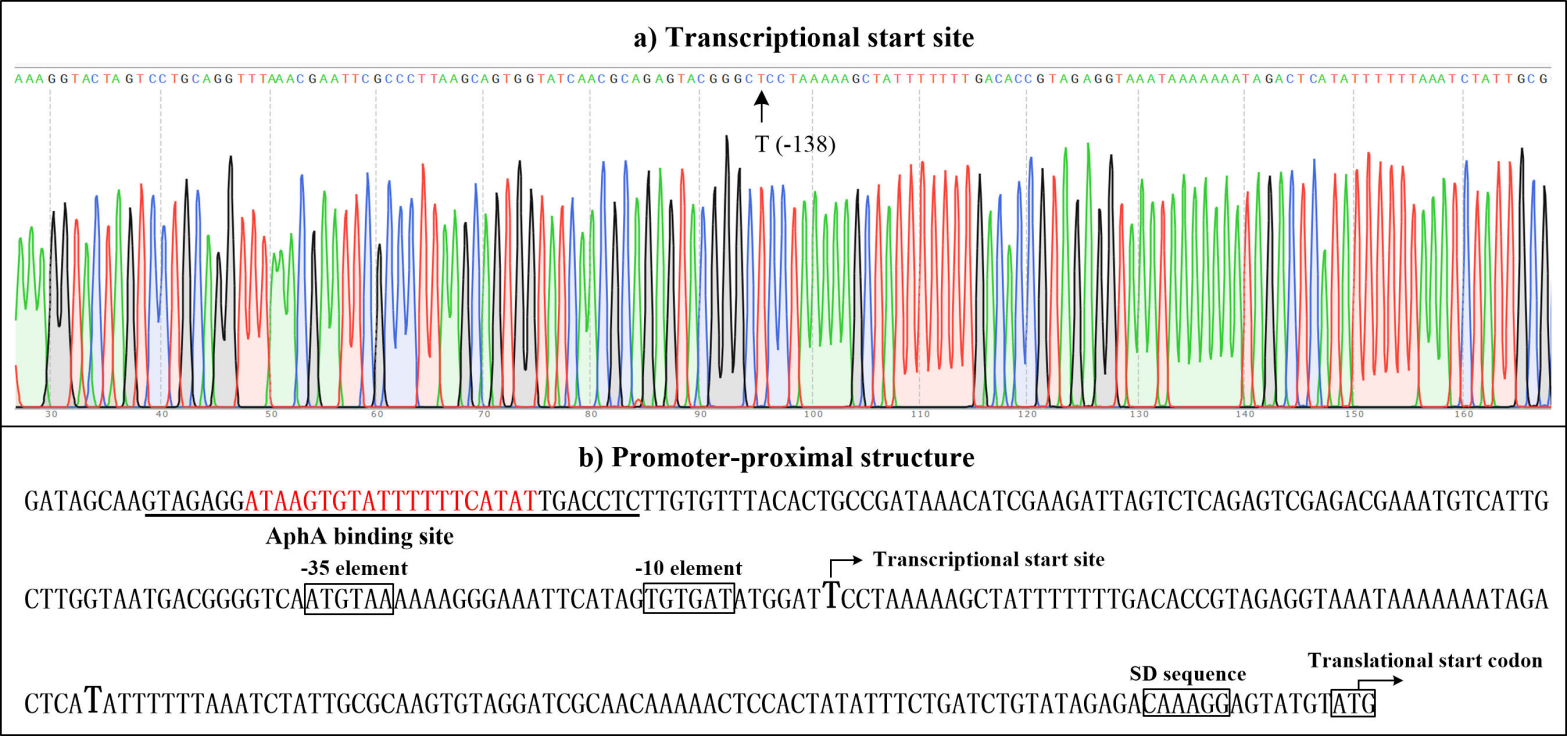
Transcriptional start site and promoter architecture of *eapA*. (a) Transcriptional start site. Negative number indicates nucleotide position upstream of the *eapA* translation start site (+1). The transcriptional start site is marked with an arrow and its corresponding position. (b) Promoter-proximal structure. The DNA sequence corresponds to *V. parahaemolyticus* RIMD2210633. Transcriptional and translational start sites are denoted by bent arrows. The Shine-Dalgarno (SD) sequence and −10/−35 promoter elements are boxed. AphA-box-like sequence is highlighted in red, and the AphA binding site is underlined with a solid line.

The transcriptional start site of *eapA* was identified using 5′-RACE (Fig. 3a). The assay revealed a single start site located 138 bp upstream of the translational start site, with the −10 and −35 promoter elements showing some similarity to the consensus prokaryotic sequences (Fig. 3b). The AphA-dependent promoter architecture of *eapA* was reconstructed by integrating data on the transcriptional start site, the −10/−35 promoter elements, the translational start codon, the AphA binding site, and the Shine-Dalgarno (SD) sequence (Fig. 3b). The AphA binding site is positioned far upstream of the −35 box, suggesting that AphA-mediated activation of *eapA* transcription likely operates via a class I transcriptional activation mechanism, which depends on interactions with the RNAP α-subunit C-terminal domain (46).

### Cell density-dependent expression of *eapA*

**Fig 4:**
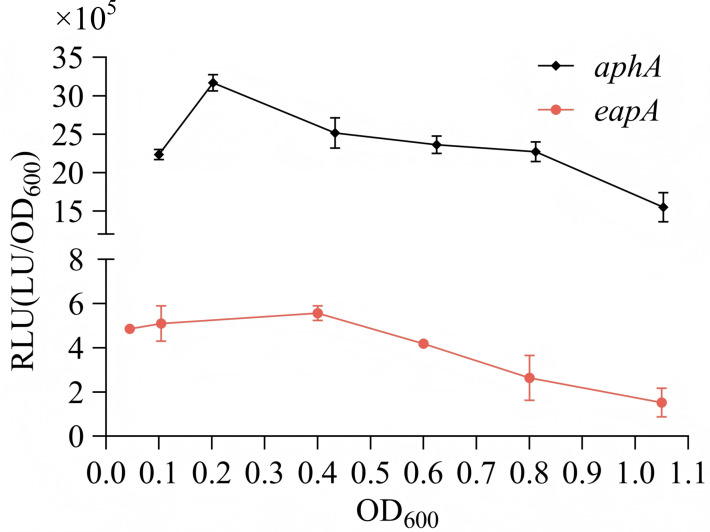
Cell density-dependent expression of *eapA*. The promoter region of *eapA* was cloned into pBBRlux and transformed into the WT strain. Cells were harvested at specified time points during growth, and luminescence was quantified using an Infinite 200 Pro NanoQuant plate reader. RLU was normalized to cell density (light units/OD_600_).

The expression of *eapA* and *aphA* during bacterial growth was monitored using a luminescence reporter assay. As shown in Fig. 4, the expression level of *aphA* increases as the OD_600_ values rise until OD_600_ reaches 0.2; beyond this point, *aphA* expression gradually decreases. This trend partially contradicts prior observations that *aphA* mRNA levels steadily decline from LCD to HCD (40, 42). This discrepancy may arise from methodological differences: the previous study measured mRNA directly, whereas our work indirectly assessed *aphA* promoter activity using a report gene. Additionally, *eapA* expression gradually decreased as cell density increased (OD_600_ ranged from 0.4 to 1.1). Its highest expression occurred below OD_600_ values below 0.4, aligning with the expression pattern of *aphA* and supporting the role of AphA in activating *eapA* transcription (Fig. 2).

### EapA and AphA modulate c-di-GMP production at LCD

**Fig 5:**
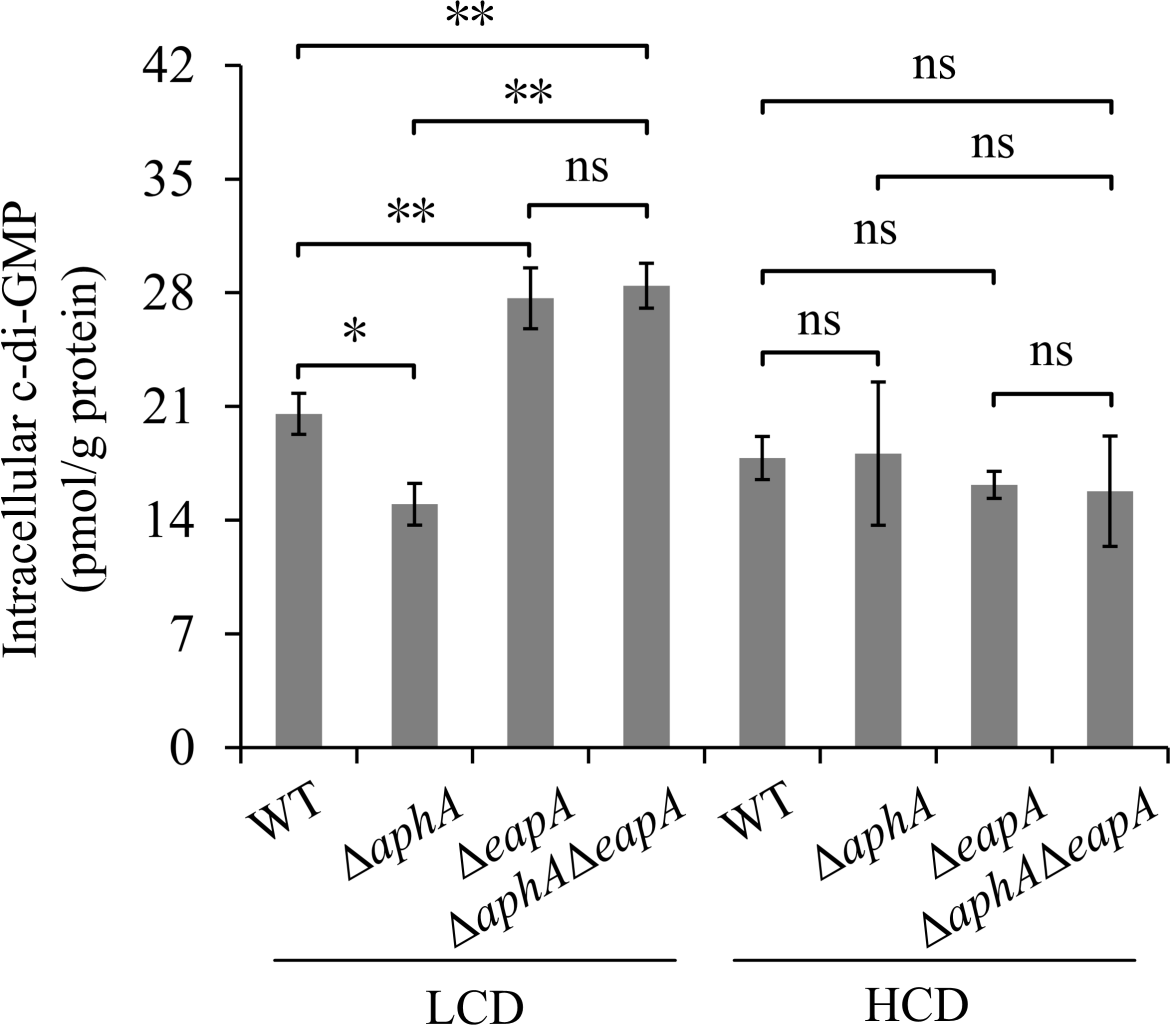
EapA and AphA modulate c-di-GMP production at LCD. Bacterial cells were harvested at OD_600_ values of 0.15 and 1.0, representing LCD and HCD conditions, respectively. Intracellular c-di-GMP concentrations were quantified using a c-di-GMP enzyme-linked immunosorbent assay (ELISA) kit. Data represent means ± SD from four biological replicates. *, *P* < 0.05; ns, *P* > 0.05.

EapA contains an EAL domain (47) and is highly expressed at LCD (Fig. 4). To assess whether EapA contributes to c-di-GMP metabolism, intracellular c-di-GMP levels were quantified in WT, Δ*aphA*, Δ*eapA*, and Δ*aphA*Δ*eapA* strains under LCD and HCD conditions. The Δ*aphA* strain was included as a control, as AphA has been previously shown to promote c-di-GMP production at LCD (31). At LCD, c-di-GMP levels in the Δ*eapA* strain were significantly higher than in the WT strain (*P* < 0.01), whereas levels in the Δ*aphA* strain were markedly reduced (*P* < 0.05; Fig. 5). Additionally, c-di-GMP levels in the Δ*aphA*Δ*eapA* strain were significantly higher than in both the WT and Δ*aphA* strains (*P* < 0.01), but there was no statistical difference between the Δ*aphA*Δ*eapA* strain and the Δ*eapA* strain (*P* > 0.05). In contrast, no significant differences in c-di-GMP levels were observed among the WT, Δ*aphA*, Δ*eapA*, and Δ*aphA*Δ*eapA* strains at HCD (*P* > 0.05). These results indicate that, at LCD, AphA and EapA exert significant and antagonistic regulatory effects on c-di-GMP metabolism. EapA likely degrades c-di-GMP, whereas AphA positively regulates its synthesis. The role of EapA in degrading c-di-GMP appears dominant, and AphA’s function is masked in the absence of EapA.

### Deletion of *eapA* enhances biofilm formation

**Fig 6:**
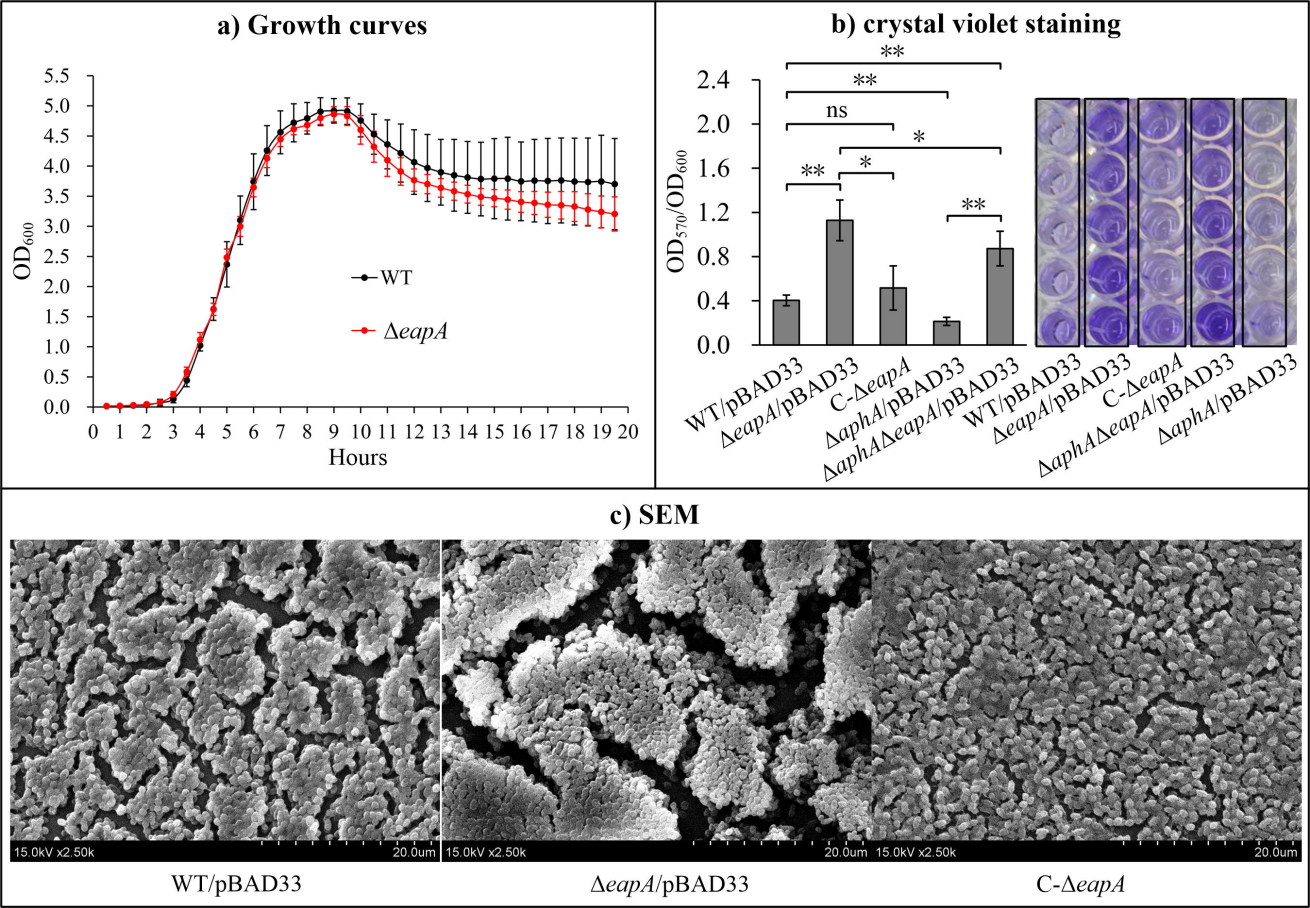
Regulation of growth and biofilm formation by EapA (and AphA). Growth curves (a) were generated by detecting OD_600_ values of bacterial cultures every 30 min using a microbial growth curve analyzer. Biofilm formation in WT/pBAD33, Δ*eapA*/pBAD33, C-Δ*eapA*, Δ*aphA*/pBAD33, and Δ*aphA*Δ*eapA*/pBAD33 was quantified by crystal violet staining (b). Biofilm architecture was visualized by SEM (c). Photographs in panels (b) and (c) are representative of three repeats. **, *P* < 0.01; *, *P* < 0.05; ns, *P* > 0.05.

Growth curves of WT and Δ*eapA* in HI broth at 37°C were determined to assess the role of EapA in bacterial growth. As shown in Fig. 6a, no differences in growth rates were observed between the two strains at any time point, suggesting that EapA does not affect *V. parahaemolyticus* growth under the tested growth conditions. The CV staining results showed that the biofilm formation ability of Δ*eapA*/pBAD33 was significantly higher than that of WT/pBAD33 and C-Δ*eapA*, while there was no significant difference between WT/pBAD33 and C-Δ*eapA* (*P* < 0.05; Fig. 6b). Furthermore, the biofilm formation ability of Δ*aphA*/pBAD33 was significantly lower than that of WT/pBAD33 (*P* < 0.01; Fig. 6b); this finding is consistent with the previous study (48). However, the biofilm formation ability of Δ*aphA*Δ*eapA*/pBAD33 was significantly higher than that of WT/pBAD33 and Δ*aphA*/pBAD33, but lower than that of Δ*eapA*/pBAD33 (*P* < 0.05; Fig. 6b). These results indicate that EapA inhibits biofilm formation, while AphA promotes biofilm formation, and the absence of *eapA* partially compensates for the impaired biofilm formation caused by the absence of *aphA*. SEM analysis showed that the WT/pBAD33, Δ*eapA*/pBAD33, and C-Δ*eapA* strains formed dense biofilm structures (Fig. 6c). Notably, biofilms formed by Δ*eapA*/pBAD33 exhibited more pronounced voids and larger cracks, suggesting a “drier” biofilm morphology with increased matrix polysaccharide content. These findings indicate that deletion of *eapA* enhances biofilm formation in *V. parahaemolyticus*.

### Deletion of *eapA* reduces swimming motility but does not affect swarming motility

**Fig 7:**
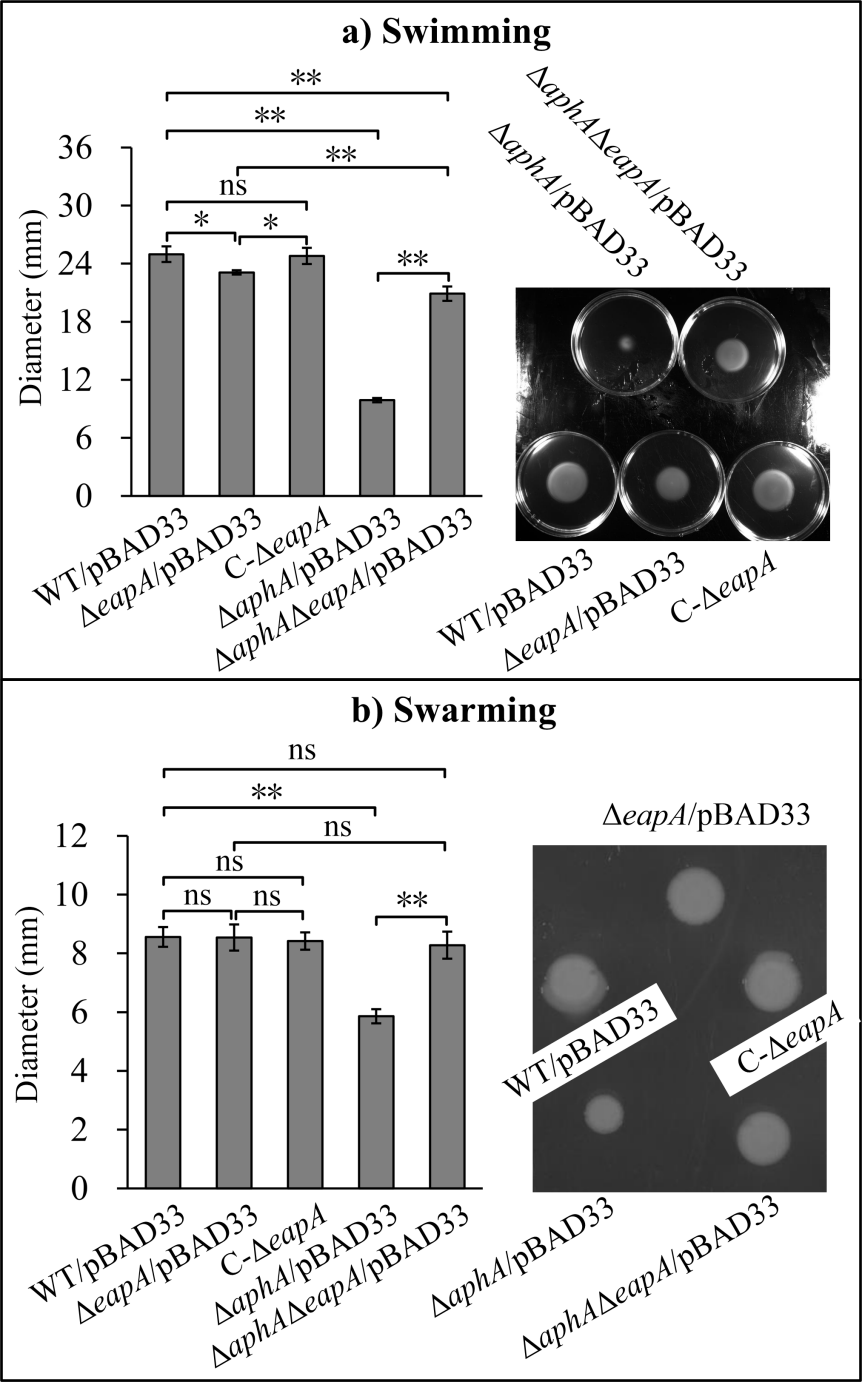
Regulation of bacterial motility by EapA and AphA. Swimming (a) and swarming (b) motility were assessed by measuring motility zone diameters for WT/pBAD33, Δ*eapA*/pBAD33, C-Δ*eapA*, Δ*aphA*/pBAD33, and Δ*aphA*Δ*eapA*/pBAD33. Photographs are representative of three replicates. **, *P* < 0.01; *, *P* < 0.05; ns, *P* > 0.05.

The swimming motility of the Δ*eapA*/pBAD33 strain was statistically lower than those of the WT/pBAD33 and C-Δ*eapA* strains (*P* < 0.05), while there was no significant difference between the WT/pBAD33 and C-Δ*eapA* strains (*P* > 0.05; Fig. 7a). Additionally, the swimming motility of the Δ*aphA*/pBAD33 strain was significantly lower than that of the WT/pBAD33 strain (*P* < 0.01; Fig. 7a), a finding consistent with previous research (48). However, the swimming motility of the Δ*aphA*Δ*eapA*/pBAD33 strain was statistically lower than those of the WT/pBAD33 and the Δ*eapA*/pBAD33 strains (*P* < 0.01), but significantly higher than that of the Δ*aphA*/pBAD33 (*P* < 0.01; Fig. 7a). In contrast, no statistical differences in swarming motility were observed among the Δ*eapA*/pBAD33, C-Δ*eapA*, WT/pBAD33, and Δ*aphA*Δ*eapA*/pBAD33 strains (*P* > 0.05; Fig. 7b). The swarming motility of the Δ*aphA*/pBAD33 strain was significantly lower than that of the WT/pBAD33 (*P* < 0.01; Fig. 7b), also consistent with previous findings (48). These results indicate that EapA promotes swimming motility in *V. parahaemolyticus* but does not affect swarming motility. In contrast, AphA promotes both swimming and swarming motility. Furthermore, EapA partially compensates for the impaired swimming motility caused by the absence of *aphA*, revealing a functional interaction between these two proteins in motility control.

### Deletion of *eapA* upregulates EPS-associated genes while downregulating polar flagellar genes

**Fig 8:**
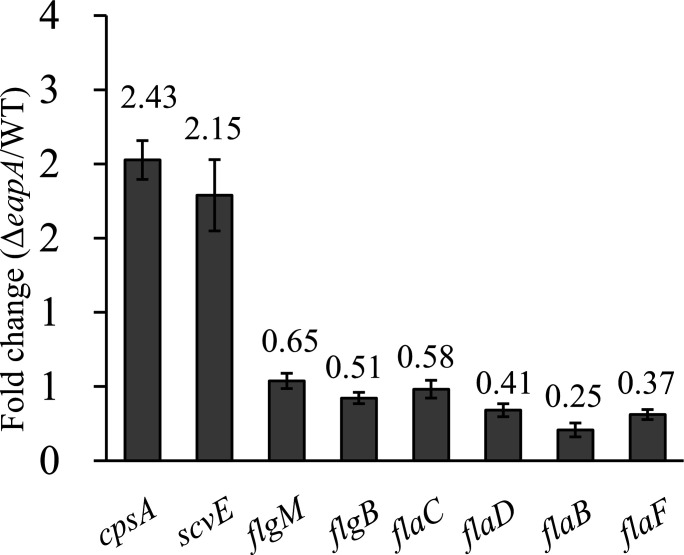
Regulation of EPS- and polar flagellum-associated genes by EapA. Bacterial cells were harvested from LCD cultures (OD_600_ ≈ 0.15). Relative mRNA levels of EPS- and polar flagellum-related genes in the Δ*eapA* strain were compared to those in the WT strain. Gene expression was normalized to the *recA* gene, which served as the internal control. Data are presented as the mean ± SD from three biological replicates. The value above each bar indicates the fold change in expression relative to the WT strain.

In this study, polar flagellar genes (*flgB*, *flgM*, *flaB*, *flaC*, *flaD*, and *flaF*) and EPS-associated genes (*cpsA* and *scvE*) were selected as targets for qRT-PCR to assess EapA-mediated regulation. As shown in Fig. 8, the mRNA levels of *flgB*, *flgM*, *flaB*, *flaC*, *flaD*, and *flaF* were downregulated to 0.51-, 0.65-, 0.25-, 0.58-, 0.41-, and 0.37-fold of their original levels, respectively, in the Δ*eapA* strain compared to the WT strain. In contrast, *cpsA* and *scvE* expression was upregulated by 2.43- and 2.15-fold in Δ*eapA*. These results indicate that EapA positively regulates polar flagellar genes while repressing EPS-related genes in *V. parahaemolyticus*.

## DISCUSSION

AphA orchestrates diverse processes in *V. parahaemolyticus*, including motility, biofilm formation, and virulence (48). It indirectly represses operons associated with T6SS1, T6SS2, and genes within Vp-PAI, while inducing T3SS1 genes via the *exsBAD-vscBCD* operon (28, 40, 43). AphA also enhances *in viv*o fitness within the mouse intestine and activates transcription of several lateral flagellar operons (41, 49). Furthermore, AphA promotes biofilm formation by regulating genes such as *mfpABC*, *scvE*, *mshA1*, and *cpsA* and acts as a direct positive regulator of the *ectABC-asp_ect* operon, which encodes biosynthetic enzymes (42, 45, 50, 51). Importantly, AphA modulates c-di-GMP levels, a key regulator of the biofilm-motility switch (14), at least partially by influencing the expression of genes like *scrABC*, *scrG*, *vpa0198*, and *vpa1477-gefB* (17, 21, 31). RNA-seq analysis in this study revealed that AphA regulates 1,542 genes involved in diverse cellular pathways (Fig. 1), supporting its role as a global regulator in *V. parahaemolyticus*.

A prior study established that AphA promotes c-di-GMP accumulation at LCD (31), but the mechanism remained elusive. Here, RNA-seq revealed that 23 c-di-GMP metabolism-associated genes were regulated by AphA at LCD (Table S2), but only *eapA* was prioritized due to its high-confidence AphA-binding motif and regulatory significance (Table 2). EMSA and DNase I footprinting confirmed that AphA directly binds the promoter region of *eapA* encoding an EAL domain-containing PDE that degrades c-di-GMP (Fig. 2). The AphA-binding site in the *eapA* promoter (−288 to −255 bp), coupled with *eapA*’s significant downregulation in the Δ*aphA* strain (Fig. 2), confirms AphA as a direct activator of *eapA*. The positioning of the AphA-binding site far upstream of the −35 element (Fig. 3b) suggests a class I activation mechanism involving RNAP α-CTD interactions (52).

The inverse relationship between *eapA* expression and c-di-GMP levels at LCD, as evidenced by elevated c-di-GMP in the Δ*eapA* strain, is consistent with a role as a functional PDE (Fig. 5). While *V. parahaemolyticus* encodes numerous confirmed PDEs, including ScrC (53), ScrG (16), GepA (54), TpdA (22), and VopY (23), EapA is the first LCD-specific PDE directly governed by AphA. The inverse correlation between *eapA* expression and cell density (Fig. 4) aligns with LCD-specific activity of AphA (26). c-di-GMP levels are usually higher at LCD than at HCD (55). AphA-mediated *eapA* activation enhances PDE activity, tilting the c-di-GMP balance toward degradation. This model is corroborated by the fact that Δ*eapA* exhibited hyper-accumulation of c-di-GMP at LCD, while Δ*aphA* showed reduced c-di-GMP (Fig. 5). Crucially, the Δ*aphA*Δ*eapA* strain had c-di-GMP levels comparable to Δ*eapA* at LCD, indicating that EapA acts downstream of AphA and is epistatic to AphA for c-di-GMP degradation.

c-di-GMP is a master regulatory factor of the biofilm-motility switch (14). Consistent with this, the deletion of *eapA* (resulting in high c-di-GMP) enhanced biofilm formation (Fig. 6) and reduced swimming motility (Fig. 7a), while the *aphA* deletion (low c-di-GMP) reduced both biofilm formation and motility (Fig. 6b and 7), as also reported previously (48). The partial restoration of biofilm formation and swimming in the Δ*aphA*Δ*eapA* strain (Fig. 6b and 7a) confirms that EapA is a key effector mediating AphA’s control over these phenotypes. The upregulation of EPS-associated genes (*cpsA* and *scvE*) and downregulation of polar flagellar genes (such as *flgB* and *flaC*) in Δ*eapA* further support the paradigm that c-di-GMP inversely regulates motility and biofilm matrix production (9, 14). Notably, the lack of impact on swarming motility suggests that lateral flagella, critical for surface movement, may be governed by distinct regulatory circuits, possibly involving other c-di-GMP metabolizing enzymes or QS components (10, 41).

The AphA-*eapA*-c-di-GMP regulatory circuit likely equips *V. parahaemolyticus* to adapt to environmental niches. At LCD, AphA and EapA are highly expressed; AphA positively regulates c-di-GMP production and *eapA* expression, while EapA degrades c-di-GMP. This may prevent excessive accumulation, maintaining c-di-GMP at reasonable levels that promote surface attachment and biofilm formation. Such dynamic regulation aligns with the ecological need for *V. parahaemolyticus* to transition between planktonic and biofilm states in response to environmental cues. While this study focuses on *eapA*, RNA-seq data revealed additional c-di-GMP-related genes (e.g., VP1979, VP2366, and VPA0518) with putative AphA-binding sites, albeit with lower confidence scores. Investigating these candidates could uncover auxiliary pathways fine-tuning c-di-GMP levels. Additionally, the proposed class I activation mechanism for AphA, which is mediated via RNA polymerase α-subunit interactions, warrants experimental validation. Whether the AphA-EapA axis influences infection outcomes requires further investigation.

In summary, this study establishes AphA as a key regulator of c-di-GMP metabolism through direct activation of *eapA*, thereby modulating the biofilm-motility switch in *V. parahaemolyticus*. These findings deepen our understanding of how QS integrates with second-messenger signaling to optimize bacterial fitness in dynamic environments. Future work exploring the interplay between AphA, other c-di-GMP metabolizing enzymes, and downstream effectors will further illuminate the complex regulatory networks governing *V. parahaemolyticus* pathogenicity and environmental persistence.

## Data Availability

The original data are available in the article or supplementary materials. RNA-seq raw data have been deposited in the NCBI repository under accession number PRJNA1235461.

## SUPPLEMENTAL MATERIAL

Table S1 jb.00104-25-s0001.xlsx AphA regulon (all DEGs).

Table S2 jb.00104-25-s0002.doc Selected DEGs.

ASM does not own the copyrights to Supplemental Material that may be linked to, or accessed through, an article. The authors have granted ASM a non-exclusive, world-wide license to publish the Supplemental Material files. Please contact the corresponding author directly for reuse.
