# Supplementary material for: AphA-dependent c-di-GMP production in Vibrio parahaemolyticus is mediated by direct regulation of eapA transcription encoding an EAL domain-containing protein
Source: J Bacteriol. 2025 Sep 18;207(10):e00104-25. doi: 10.1128/jb.00104-25 (PMC12548387; doi:10.1128/jb.00104-25)
Supplement: Table S2 — Selected DEGs. [file jb.00104-25-s0002.doc]

**Table S2. Selected DEGs**

| **Locus** | **Name** | **Fold change** | **Products** |
| --- | --- | --- | --- |
| **c-di-GMP** | | | |
| VP0117 | *gepA* | 0.4745 | EAL domain-containing protein |
| VP0376 | *eapA* | 0.3729 | EAL domain-containing protein |
| VP0486 |  | 0.0015 | diguanylate cyclase |
| VP1427 |  | 0.1397 | c-di-GMP phosphodiesterase |
| VP1637 |  | 2.0583 | GGDEF domain-containing protein |
| VP1754 |  | 2.7252 | EAL domain-containing protein |
| VP1768 |  | 0.4869 | EAL domain-containing protein |
| VP1881 | *tpdA* | 5.4081 | EAL domain-containing protein |
| VP1979 |  | 0.3975 | EAL domain-containing protein |
| VP2366 |  | 0.3576 | sensor domain-containing diguanylate cyclase |
| VP2708 | *csrD* | 0.3998 | RNase E specificity factor CsrD |
| VP2888 |  | 2.1505 | sensor domain-containing diguanylate cyclase |
| VPA0059 |  | 0.1785 | GGDEF domain-containing protein |
| VPA0184 |  | 0.4170 | GGDEF domain-containing protein |
| VPA0476 |  | 0.2609 | sensor domain-containing diguanylate cyclase |
| VPA0518 |  | 0.4216 | GGDEF domain-containing phosphodiesterase |
| VPA0609 |  | 0.2737 | bifunctional diguanylate cyclase/phosphodiesterase |
| VPA0869 |  | 0.1747 | GGDEF and EAL domain-containing protein |
| VPA0878 |  | 2.7076 | diguanylate cyclase |
| VPA0927 |  | 0.4101 | diguanylate cyclase |
| VPA1324 |  | 12.6735 | EAL domain-containing protein |
| VPA1429 |  | 0.4117 | PTS sugar transporter subunit IIC/EAL domain-containing protein |
| VPA1457 |  | 0.3842 | GGDEF domain-containing protein |
| **Flagella** | | | |
| VP0770 | *flgN* | 0.4664 | flagellar export chaperone FlgN |
| VP0771 | *flgM* | 0.3836 | flagellar biosynthesis anti-sigma factor FlgM |
| VP0775 | *flgB* | 0.2026 | flagellar basal body rod protein FlgB |
| VP0776 | *flgC* | 0.1471 | flagellar basal body rod protein FlgC |
| VP0777 | *flgD* | 0.1340 | flagellar hook assembly protein FlgD |
| VP0778 | *flgE* | 0.1501 | flagellar hook protein FlgE |
| VP0780 |  | 0.1466 | flagellar basal body rod protein FlgF |
| VP0781 | *flgG* | 0.0550 | flagellar basal-body rod protein FlgG |
| VP0782 | *flgH* | 0.0808 | flagellar basal body L-ring protein FlgH |
| VP0783 |  | 0.1187 | flagellar basal body P-ring protein FlgI |
| VP0784 | *flgJ* | 0.0975 | flagellar assembly peptidoglycan hydrolase FlgJ |
| VP0785 | *flgK* | 0.1167 | flagellar hook-associated protein FlgK |
| VP0786 | *flgL* | 0.1556 | flagellar hook-associated protein FlgL |
| VP0788 |  | 0.1722 | flagellin |
| VP0790 |  | 0.0509 | flagellin |
| VP0791 |  | 0.1681 | flagellin |
| VP2111 |  | 0.2385 | OmpA family protein |
| VP2225 |  | 0.4423 | chemotaxis protein CheW |
| VP2227 |  | 0.4464 | ParA family protein |
| VP2228 |  | 0.4617 | chemotaxis response regulator protein-glutamate methylesterase |
| VP2229 |  | 0.3654 | chemotaxis protein CheA |
| VP2233 |  | 0.4037 | MinD/ParA family protein |
| VP2234 | *flhF* | 0.3270 | flagellar biosynthesis protein FlhF |
| VP2235 | *flhA* | 0.4479 | flagellar biosynthesis protein FlhA |
| VP2238 | *fliQ* | 0.2775 | flagellar biosynthesis protein FliQ |
| VP2241 | *fliN* | 0.3654 | flagellar motor switch protein FliN |
| VP2242 | *fliM* | 0.4541 | flagellar motor switch protein FliM |
| VP2244 |  | 0.2924 | flagellar hook-length control protein FliK |
| VP2245 | *fliJ* | 0.4424 | flagella biosynthesis chaperone FliJ |
| VP2247 | *fliH* | 0.4570 | flagellar assembly protein FliH |
| VP2248 | *fliG* | 0.3914 | flagellar motor switch protein FliG |
| VP2249 | *fliF* | 0.4722 | flagellar M-ring protein FliF |
| VP2251 |  | 0.3057 | sigma-54 dependent transcriptional regulator |
| VP2252 |  | 0.4603 | PAS domain-containing protein |
| VP2254 | *fliS* | 0.1046 | flagellar export chaperone FliS |
| VP2255 |  | 0.0073 | flagellar protein FliT |
| VP2256 | *fliD* | 0.1330 | flagellar filament capping protein FliD |
| VP2257 | *flaG* | 0.1049 | flagellar protein FlaG |
| VP2258 |  | 0.0848 | flagellin |
| VP2259 |  | 0.0588 | flagellin |
| VP2261 |  | 0.1200 | flagellin |
| VP2811 |  | 0.1439 | sel1 repeat family protein |
| VPA1536 | *fliF* | 0.4482 | flagellar M-ring protein FliF |
| VPA1537 | *fliE* | 0.0924 | flagellar hook-basal body complex protein FliE |
| VPA1538 |  | 0.3044 | sigma-54 dependent transcriptional regulator |
| VPA1539 |  | 0.1979 | OmpA family protein |
| VPA1540 |  | 0.2764 | flagellar motor switch protein FliM |
| **CPS** | | | |
| VP0216 |  | 2.3334 | capsule biosynthesis GfcC family protein |
| VP0217 |  | 2.4343 | YjbF family lipoprotein |
| VP0221 |  | 2.1899 | LPS biosynthesis protein |
| VP0224 |  | 2.5429 | sugar nucleotide-binding protein |
| VP0226 |  | 3.3841 | glycosyltransferase family 2 protein |
| VP0227 |  | 2.9843 | hypothetical protein |
| VP0228 |  | 2.3902 | oligosaccharide repeat unit polymerase |
| VP0230 |  | 3.2450 | glycosyltransferase family 4 protein |
| VP0231 |  | 2.2081 | sugar transferase |
| VP0232 |  | 2.0837 | ATP-grasp domain-containing protein |
| VP0233 |  | 2.0485 | HAD hydrolase-like protein |
| **EPS** | | | |
| VP1458 |  | 0.1561 | sugar transferase |
| VP1459 |  | 0.3090 | glycosyltransferase family 2 protein |
| VP1461 |  | 0.1304 | glycosyltransferase |
| VP1468 |  | 0.0357 | glycosyltransferase family 4 protein |
| VP1469 |  | 0.0107 | sigma-54 dependent transcriptional regulator |
| VP1472 |  | 0.0631 | Hpt domain-containing protein |
| VP1473 |  | 0.1136 | CpsD/CapB family tyrosine-protein kinase |
| VP1474 |  | 0.0337 | SLBB domain-containing protein |
| VP1475 |  | 0.0302 | OmpA family protein |
| **T3SS1** | | | |
| VP1656 | *vopD* | 0.2171 | type III secretion system translocon subunit VopD |
| VP1657 | *vopB* | 0.2594 | type III secretion system translocon subunit VopB |
| VP1658 | *vcrH* | 0.3790 | SycD/LcrH family type III secretion system chaperone VcrH |
| VP1659 | *vcrV* | 0.3275 | type III secretion system needle tip protein VcrV |
| VP1660 | *vcrG* | 0.4585 | LcrG family type III secretion system chaperone VcrG |
| VP1664 | *vscX* | 0.2250 | type III secretion system protein VscX |
| VP1665 | *sycN* | 0.2888 | type III secretion chaperone SycN |
| VP1666 |  | 0.3211 | TyeA family type III secretion system gatekeeper subunit |
| VP1667 | *vopN* | 0.1964 | SctW family type III secretion system gatekeeper subunit VopN |
| VP1668 | *vscN* | 0.3520 | SctN family type III secretion system ATPase VscN |
| VP1676 |  | 2.0584 | LysR family transcriptional regulator |
| VP1680 | *vopQ* | 0.1188 | type III secretion system effector VopQ |
| VP1682 | *vecA* | 0.1837 | CesT family type III secretion system chaperone VecA |
| VP1683 | *vopR* | 0.4039 | type III secretion system effector VopR |
| VP1686 | *vopS* | 0.2847 | T3SS effector adenosine monophosphate-protein transferase VopS |
| VP1687 |  | 0.3196 | CesT family type III secretion system chaperone |
| VP1690 | *vscJ* | 0.4313 | SctJ family type III secretion inner membrane ring lipoprotein Vsc |
| VP1692 | *vscH* | 0.4633 | YopR family T3SS polymerization control protein VscH |
| VP1693 | *vscG* | 0.3300 | YscG family type III secretion system chaperone VscG |
| VP1694 | *vscF* | 0.3562 | type III secretion system needle filament protein VscF |
| VP1698 | *esxD* | 0.4253 | type III secretion system regulon anti-activator ExsD |
| VP1699 | *exsA* | 0.4982 | type III secretion system transcriptional regulator ExsA |
| VP1700 |  | 0.3626 | YscW family type III secretion system pilotin |
| VP1701 | *exsC* | 0.1243 | type III secretion system regulatory chaperone ExsC |
| VP1702 | *exsE2* | 0.2825 | T3SS regulon translocated regulator ExsE2 |
| VP2526 |  | 2.4997 | A24 family peptidase |
| VP2695 |  | 0.4601 | type II secretion system GspH family protein |
| VP2696 |  | 0.4686 | type II secretion system GspH family protein |
| VP2699 |  | 0.4394 | MSHA biogenesis protein MshF |
| **T6SS** | | | |
| VP1414 | *icmH* | 4.2444 | type IVB secretion system protein IcmH/DotU |
| VP1416 |  | 2.0972 | hypothetical protein |
| VP1419 |  | 3.4109 | hypothetical protein |
| VPA1027 |  | 2.1017 | type VI secretion system tube protein Hcp |
| VPA1033 | *tssC* | 5.2594 | type VI secretion system contractile sheath large subunit |
| VPA1036 | *tssA* | 4.7422 | type VI secretion system protein TssA |
| VPA1037 |  | 2.8878 | protein phosphatase 2C domain-containing protein |
| VPA1038 | *tagF* | 4.0866 | type VI secretion system-associated protein TagF |
| VPA1039 | *tssM* | 2.4591 | type VI secretion system membrane subunit TssM |
| **T3SS2** | | | |
| VPA1321 | *vopC* | 5.4222 | T3SS2 effector GTPase-activating deamidase VopC |
| VPA1322 |  | 6.4013 | hypothetical protein |
| VPA1323 |  | 4.8034 | hypothetical protein |
| VPA1326 |  | 10.5160 | hypothetical protein |
| VPA1327 | *vopT* | 27.2289 | T3SS effector ADP-ribosyltransferase toxin VopT |
| VPA1328 |  | 40.3448 | hypothetical protein |
| VPA1329 |  | 30.6576 | conjugal transfer protein TraA |
| VPA1331 |  | 26.2473 | VPA1331 family putative T3SS effector |
| VPA1334 |  | 13.8351 | hypothetical protein |
| VPA1335 |  | 12.2940 | flagellar biosynthetic protein FliQ |
| VPA1336 | *vopZ* | 24.5112 | type III secretion system effector VopZ |
| VPA1337 |  | 20.1081 | VPA1337 family putative T3SS effector |
| VPA1338 |  | 15.4105 | type III secretion system ATPase |
| VPA1339 |  | 22.4166 | secretin |
| VPA1340 |  | 23.3834 | VPA1340 family putative T3SS effector |
| VPA1341 |  | 55.1881 | hypothetical protein |
| VPA1342 |  | 44.7532 | EscR/YscR/HrcR family type III secretion system export apparatus protein |
| VPA1343 |  | 12.5990 | hypothetical protein |
| VPA1345 |  | 9.6197 | hypothetical protein |
| VPA1346 | *vopA* | 18.0343 | type III secretion system YopJ family effector VopA |
| VPA1348 | *vtrB* | 9.4356 | winged helix-turn-helix domain-containing protein |
| VPA1349 |  | 9.1981 | FliM/FliN family flagellar motor switch protein |
| VPA1350 |  | 9.6937 | VPA1350 family putative T3SS effector |
| VPA1351 |  | 18.0044 | VPA1351 family putative T3SS effector |
| VPA1352 |  | 16.8527 | VPA1352 family putative T3SS effector |
| VPA1353 |  | 15.2766 | OmpA family protein |
| VPA1354 |  | 21.8978 | EscU/YscU/HrcU family type III secretion system export apparatus switch protein |
| VPA1355 |  | 15.8431 | FHIPEP family type III secretion protein |
| VPA1356 |  | 10.9329 | hypothetical protein |
| VPA1357 |  | 16.0727 | hypothetical protein |
| VPA1358 |  | 47.7338 | dimethyladenosine transferase |
| VPA1359 |  | 28.4202 | hypothetical protein |
| VPA1360 |  | 33.2705 | hypothetical protein |
| VPA1361 | *vopD2* | 19.4584 | type III secretion system translocator protein VopD2 |
| VPA1362 | *vopB*2 | 18.3516 | type III secretion system translocator protein VopB2 |
| VPA1363 |  | 32.9859 | molecular chaperone |
| VPA1364 |  | 35.4862 | hypothetical protein |
| VPA1365 |  | 28.3212 | hypothetical protein |
| VPA1366 |  | 24.4820 | hypothetical protein |
| VPA1367 |  | 19.4203 | type III secretion protein |
| VPA1368 |  | 16.8753 | hypothetical protein |
| VPA1369 |  | 6.5360 | hypothetical protein |
| VPA1370 | *vopL* | 7.3857 | type III secretion system effector VopL |
| Regulator | | | |
| VP0067 |  | 0.2250 | LysR family transcriptional regulator |
| VP0072 | *asnC* | 0.4690 | transcriptional regulator AsnC |
| VP0118 | *glnG* | 0.4792 | nitrogen regulation protein NR(I) |
| VP0247 | *rraA* | 0.2423 | ribonuclease E activity regulator RraA |
| VP0358 | *scrO* | 3.3669 | DeoR family transcriptional regulator |
| VP0368 |  | 0.4456 | MltR family transcriptional regulator |
| VP0487 | *arcB* | 0.0007 | aerobic respiration two-component sensor histidine kinase ArcB |
| VP0489 | *arcA* | 0.0005 | two-component system response regulator ArcA |
| VP0538 | *btsR* | 0.4691 | two-component system response regulator BtsR |
| VP0635 |  | 0.2663 | LysR family transcriptional regulator |
| VP0813 |  | 0.1088 | P-II family nitrogen regulator |
| VP0914 |  | 0.2655 | sigma-54 dependent transcriptional regulator |
| VP0915 |  | 0.3451 | sensor histidine kinase |
| VP1032 | *torR* | 0.4819 | two-component system response regulator TorR |
| VP1136 |  | 0.3705 | sigma-54 dependent transcriptional regulator |
| VP1190 | *norR* | 2.7290 | nitric oxide reductase transcriptional regulator NorR |
| VP1201 |  | 0.4906 | sensor histidine kinase |
| VP1211 |  | 2.3999 | sensor histidine kinase |
| VP1236 |  | 0.4007 | MurR/RpiR family transcriptional regulator |
| VP1244 |  | 0.2640 | response regulator |
| VP1316 |  | 0.4202 | LysR family transcriptional regulator |
| VP1375 |  | 0.3475 | cyclic nucleotide-binding domain-containing protein |
| VP1376 |  | 0.2024 | response regulator |
| VP1469 |  | 0.0107 | sigma-54 dependent transcriptional regulator |
| VP1472 |  | 0.0631 | Hpt domain-containing protein |
| VP1613 |  | 2.0190 | GntR family transcriptional regulator |
| VP1649 |  | 0.2770 | GntR family transcriptional regulator |
| VP1711 |  | 0.2968 | response regulator |
| VP1712 |  | 0.4379 | sensor histidine kinase |
| VP1734 |  | 0.1775 | response regulator |
| VP1735 |  | 0.1991 | two-component sensor histidine kinase |
| VP1755 |  | 0.0832 | response regulator |
| VP1876 |  | 0.1074 | response regulator |
| VP1907 |  | 0.1391 | response regulator transcription factor |
| VP1908 |  | 0.1966 | cache domain-containing protein |
| VP1939 |  | 0.1230 | transcriptional regulator |
| VP1962 |  | 0.3122 | Crp/Fnr family transcriptional regulator |
| VP1976 | *metR* | 0.4257 | HTH-type transcriptional regulator MetR |
| VP2127 |  | 0.3713 | Hpt domain-containing protein |
| VP2165 |  | 2.7399 | ribbon-helix-helix domain-containing protein |
| VP2183 |  | 0.1631 | response regulator |
| VP2228 |  | 0.4617 | chemotaxis response regulator protein-glutamate methylesterase |
| VP2229 |  | 0.3654 | chemotaxis protein CheA |
| VP2251 |  | 0.3057 | sigma-54 dependent transcriptional regulator |
| VP2252 |  | 0.4603 | PAS domain-containing protein |
| VP2266 |  | 0.3947 | helix-turn-helix domain-containing protein |
| VP2378 |  | 0.4650 | LysR family transcriptional regulator |
| VP2424 |  | 0.0633 | AraC family transcriptional regulator |
| VP2427 |  | 0.2354 | LysR family transcriptional regulator |
| VP2752 | *oxyR* | 0.3168 | DNA-binding transcriptional regulator OxyR |
| VP2858 |  | 0.2865 | response regulator |
| VP2866 |  | 0.2601 | response regulator transcription factor |
| VP2874 |  | 0.4901 | hybrid sensor histidine kinase/response regulator |
| VP2885 | *fis* | 2.0338 | DNA-binding transcriptional regulator Fis |
| VPA0009 |  | 0.1593 | response regulator |
| VPA0020 |  | 0.3311 | sensor histidine kinase |
| VPA0021 |  | 0.3754 | LytTR family DNA-binding domain-containing protein |
| VPA0034 |  | 0.2169 | GntR family transcriptional regulator |
| VPA0073 |  | 0.4350 | LysR family transcriptional regulator |
| VPA0076 | *kdgR* | 0.4687 | DNA-binding transcriptional regulator KdgR |
| VPA0148 |  | 2.1010 | response regulator transcription factor |
| VPA0149 |  | 3.6114 | sensor histidine kinase |
| VPA0177 |  | 0.4001 | helix-turn-helix domain-containing protein |
| VPA0214 |  | 0.4918 | YafY family transcriptional regulator |
| VPA0249 |  | 3.2562 | LysR family transcriptional regulator |
| VPA0315 |  | 0.4904 | LysR family transcriptional regulator |
| VPA0331 |  | 0.3486 | LysR family transcriptional regulator |
| VPA0381 |  | 7.5813 | AraC family transcriptional regulator |
| VPA0387 |  | 0.3873 | LysR family transcriptional regulator |
| VPA0415 |  | 2.5534 | AraC family transcriptional regulator |
| VPA0497 |  | 0.2990 | winged helix-turn-helix domain-containing protein |
| VPA0602 |  | 0.1578 | LysR family transcriptional regulator |
| VPA0619 |  | 0.0437 | MerR family DNA-binding transcriptional regulator |
| VPA0662 | *cueR* | 0.4587 | Cu(I)-responsive transcriptional regulator |
| VPA0675 | *torS* | 0.4668 | TMAO reductase system sensor histidine kinase/response regulator TorS |
| VPA0717 |  | 0.3538 | LysR family transcriptional regulator |
| VPA0746 |  | 0.4706 | chemotaxis protein |
| VPA0764 |  | 0.3243 | response regulator |
| VPA0804 |  | 0.2614 | XRE family transcriptional regulator |
| VPA0912 |  | 2.0038 | LysR family transcriptional regulator |
| VPA0920 |  | 2.2569 | sensor histidine kinase |
| VPA0927 |  | 0.4101 | diguanylate cyclase |
| VPA0961 | *acsS* | 3.5761 | LysR family transcriptional regulator |
| VPA0964 | *uhpA* | 0.3652 | transcriptional regulator UhpA |
| VPA0988 | *rnk* | 2.7955 | nucleoside diphosphate kinase regulator |
| VPA1049 |  | 0.0319 | SpoIIE family protein phosphatase |
| VPA1130 |  | 0.1410 | response regulator |
| VPA1162 |  | 0.4428 | response regulator |
| VPA1178 |  | 0.3519 | sugar-binding transcriptional regulator |
| VPA1195 |  | 0.3363 | response regulator |
| VPA1219 |  | 2.2840 | MarR family transcriptional regulator |
| VPA1229 |  | 0.1578 | response regulator |
| VPA1233 |  | 0.4761 | LysR family transcriptional regulator |
| VPA1446 |  | 6.8244 | helix-turn-helix transcriptional regulator |
| VPA1447 |  | 4.6964 | LuxR family transcriptional regulator |
| VPA1472 |  | 0.3912 | MerR family transcriptional regulator |
| VPA1500 |  | 4.4464 | helix-turn-helix domain-containing protein |
| VPA1538 |  | 0.3044 | sigma-54 dependent transcriptional regulator |
| VPA1623 | *malT* | 0.3746 | HTH-type transcriptional regulator MalT |
| VPA1636 |  | 0.3422 | helix-turn-helix transcriptional regulator |
| VPA1665 |  | 0.3922 | response regulator transcription factor |
| VPA1687 |  | 0.1907 | LysR family transcriptional regulator |
| VPA1713 |  | 0.4455 | AraC family transcriptional regulator |
| VPA1732 |  | 0.4997 | sigma-54 dependent transcriptional regulator |
